# Constructing Neuron-like Structured NiS_2_/MOF Composites with Enhanced Regulation of Electron Transport and Active Sites for Oxygen Evolution

**DOI:** 10.3390/molecules30010080

**Published:** 2024-12-28

**Authors:** Yanli Guo, Di Zhou, Yanyan Huang, Xiaolong Song, Wei He

**Affiliations:** 1 School of Mechanical Engineering, Chengdu University, Chengdu 610106, China; 2 State Key Laboratory for Mechanical Behaviour of Materials, Xi’an Jiaotong University, Xi’an 710049, China; 3 School of Aeronautics and Astronautics, Sichuan Univeristy, Chengdu 610065, China

**Keywords:** pseudo-neuronal structure, metal–organic framework, nickel sulfide heteroparticles, oxygen evolution reactions

## Abstract

Constructing fast electron transfer pathways and abundant electro-active sites is an effective strategy to improve the oxygen evolution reaction (OER) performance of catalysts. Herein, structural engineering and dual-phase engineering were employed to construct a NiS_2_ nanoparticle-encapsulated MOF configured with a pseudo-neuronal structure (NiS_2_/MOF/HT). It was found that the pseudo-neuronal structure, constructed with a carbon nanohorn (CNH) and carbon nanotube (CNT), provided fast electron transfer pathways and abundant exposed active sites. Moreover, the NiS_2_/MOF/HT composite obtained via partial vulcanization not only inherited the pseudo-neuronal structure but also prevented the aggregation and growth of NiS_2_ particles. NiS_2_/MOF composites provide various active sites. With the combination of the promotion of electronic transfer and enrichment of electro-active sites (NiS_2_, MOF), NiS_2_/MOF/HT showed excellent performance, whose overpotential at 25 mA cm^−2^ was reduced by 19.5% compared with MOF/HT.

## 1. Introduction

Hydrogen energy is a promising candidate to replace fossil fuels to address the increasing energy demands and environmental issues regarding sustainability [1,2,3]. Electrochemical water splitting provides a convenient and effective means to produce high-purity hydrogen [4,5,6], which is composed of the oxygen evolution reaction (OER) and hydrogen evolution reaction (HER) [7,8,9]. In contrast to the HER, the OER is a complex multi-electronic transfer process (2H_2_O = O_2_ + 4H^+^ + 4e^−^) that exhibits sluggish kinetics, posing a higher requirement for catalysts, especially regarding conductivity [10,11,12,13]. The rapid charge and mass transfer rate in the OER process plays a very important role in improving the reaction efficiency. Reasonable structure design and composition control, enriching the catalytic centers and improving the dispersion of catalytic sites, is the key to designing efficient OER catalysts.

Recently, metal–organic frameworks (MOFs) have attracted significant attention in electrocatalysis owing to their unique tunable structures, large surface areas, and the diversity of metal nodes for improved reaction kinetics [14,15,16,17,18,19]. Moreover, MOFs can be used as self-sacrificed precursors to construct metal oxide or metal nanoparticles (NPs) [20,21,22,23,24]. These obtained MOF derivatives possess various rich active interface sites and coordinated unsaturated atomic centers, which can significantly improve the OER. Thus, MOFs and their derivatives are promising candidates for the OER, as investigated in several studies [25,26]. Specifically, transition metal sulfides (TMSs), prepared with MOFs as templates, not only inherit the structural advantages of MOFs but are also more conducive to the generation of oxygen-containing intermediates during the OER reaction, due to the higher electronegativity of the sulfur (S) element in TMSs, which can obtain electrons from transition metal atoms [27]. Previous studies on catalysts for the OER have focused on pure derivative catalysts that originated from MOFs. Unfortunately, they show poor conductivity and low thermal stability, which greatly limit the catalytic activity of MOFs and bring a series of problems in the preparation of MOF derivatives, such as the collapse of the MOF skeleton structure and the aggregation of the derivative. These need to be examined. Constructing composites, such as MOFs and MOF derivatives, may be a good solution to the above problems and can be considered a promising approach toward an enhanced OER [28]. 

Herein, structural engineering and dual-phase engineering were used to construct a composite for the OER. Introducing carbon-based carriers such as CNTs and CNHs to MOFs can enhance their electrical conductivity and structural stability at high temperatures [29,30]. However, simple physical combination, rather than clever structural design, results in a limited improvement. In order to further improve the electrical chemistry performance of MOF-based catalysts, it is better to improve both the electron transport efficiency and reaction efficiency through structural design. Neurons have efficient transmission and processing capabilities, benefiting from their tree-like structures, which are composed of dendrites, an axon, and a cell body. Inspired by this, we propose herein novel carbon-based carriers with neuronal structures to enhance the complex four-electron transfer and overcome the poor conductivity and thermal stability of MOFs. In detail, a three-dimensional (3D) conductive network with a neuronal structure (named HT) was fabricated by combining CNHs and CNTs using a bionic organic binder, polydopamine (PDA). Then, an MOF network was further coated on the surface of the HT and a composite catalyst (named MOF/HT) was constructed. 

Utilizing composites containing MOFs and MOF derivatives is an effective means to improve the OER performance [15,28]. Herein, the MOF/HT was further used as a template and precursor to prepare NiS_2_/MOF/HT composite catalysts (named MOF/HT-S) with a uniform distribution and controllable morphology through controlled partial atmosphere vulcanization. Then, the MOF/HT-S was synthesized in situ on the surface of the conductive network, serving as the active site. This method can effectively prevent the aggregation and growth of NiS_2_ particles and ensure the dispersion of active sites. Moreover, it inherits the three-dimensional pseudo-neuronal structure of the MOF/HT and improves the conductivity and surface area of the used catalysts.

## 2. Results and Discussion

### 2.1. Fabrication Strategy

**Scheme 1:**
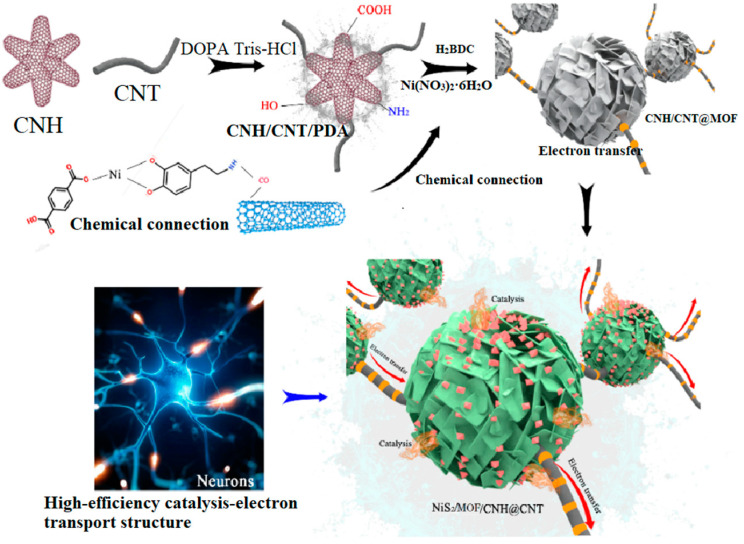
Fabrication mechanism of analog neuromorphic NiS_2_/CNH@CNT.

A neuron-like structured NiS_2_/MOF/HT catalyst was prepared by the binding of CNHs and CNTs (HT), the in situ synthesis of an MOF on the HT, and controlled partial vulcanization. Specifically, CNHs and CNTs were first bonded to form a conductive substrate network using biomimetic PDA, owing to its strong adhesion and widely available nature [26,27]. Next, an MOF network was fabricated on the HT surface through hydrothermal reactions. According to previous research, PDA not only connects CNHs to CNTs but also directs the growth of MOFs on the HT surface, forming a well-defined core–shell structure [26]. Furthermore, NiS_2_/MOF/HT with a hierarchical structure was obtained through controlled partial vulcanization in a sulfur atmosphere. The detailed fabrication mechanism is shown in Scheme 1.

### 2.2. Preparation and Characterization of MOF/HT

#### 2.2.1. Characterization of MOF/HT

**Figure 1:**
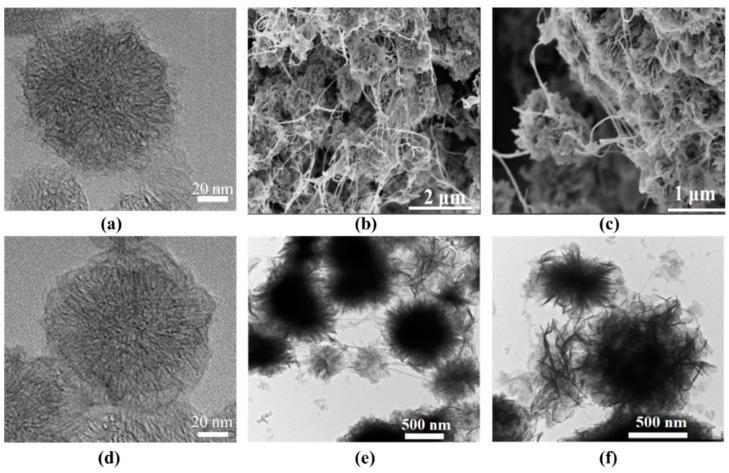
TEM images of CNH (**a**), CNH@PDA (**d**), and MOF/HT (**e**,**f**) and SEM images of MOF/HT (**b**,**c**).

The morphology of the as-prepared samples was characterized by TEM and SEM (Figure 1). As shown in Figure 1a, the CNHs agglomerated into a typical “dahlia-like” structure consisting of aggregates of shorter nanohorns. After modification with PDA, a visible nanofilm was observed, and the surface of the CNH@PDA appeared smooth. The TEM and SEM images of the MOF/HT (Figure 1b,c,e,f) revealed a typical neuronal-like structure, where the MOF was supported by the CNHs and connected by the CNTs. In this neuronal-like structure, the cyton MOF/CNH exhibited an echinoid-like and flower-like configuration, which facilitated the exposure of active sites. Additionally, dendritic CNTs extended outward and connected the cyton MOF/CNH, promoting charge and mass transport during the OER process. 

**Figure 2:**
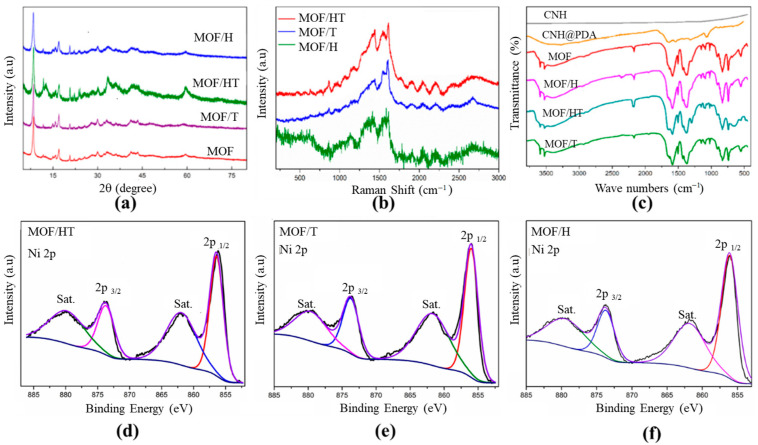
XRD patterns (**a**) and Raman spectra (**b**) of different as-prepared different materials; FTIR spectra for different samples (**c**); and Ni 2p XPS for MOF/HT (**d**), MOF/T (**e**), and MOF/H (**f**) (For **d**–**f**, The black curve is the experimentally measured data, and the color curve is the mathematical fit to it).

An XRD analysis was performed to determine the phase and crystal structures of the as-prepared samples. As shown in Figure 2a, the characteristic diffraction peaks of the as-prepared MOF corresponded well with those reported previously [26], confirming the successful synthesis of the NiMOF in this study. After introducing various carbon-based carriers, the XRD patterns of the catalysts with different carbon substrates, such as MOF/HT, MOF/H, and MOF/T, closely matched those of the pure NiMOF, indicating the successful deposition of the MOF films on the surfaces of the different carbon matrix materials (HT, CNH, and CNT) (Figure 2a). Notably, the characteristic peaks of the MOF/carbon-based carriers were slightly broader than those of the pure MOF. This was likely due to the lattice distortion of the MOF caused by the incorporation of carbon materials. Furthermore, the diffraction peaks of the MOF/HT were the broadest, indicating that the lattice distortion induced by the HT with a pseudo-neuronal structure was more pronounced than that of the mono-component. The higher defect density in the MOF/HT also likely affected its catalytic performance. These results confirm the successful fabrication of hybrid MOF/carbon-based carriers and highlight the more significant lattice distortion caused by the HT.

Raman spectroscopy was used to investigate the structural properties of different carbon materials. As shown in Figure 2b, the Raman spectra of the MOF/HT, MOF/T, and MOF/H exhibit two prominent characteristic peaks at ~1348 and 1578 cm^−1^, corresponding to the D- and G-bands of carbon materials, respectively. The D-band represents the defect and disorder density in the carbon materials, while the G-band indicates the vibration of ordered sp^2^ hybridized carbon. In the Raman spectrum of NiMOF/T, the G-band is dominant, with a relatively weak D-band. Additionally, the G-band splits into a shoulder peak, which is attributed to the high content of sp^2^ hybridized carbon in CNTs, indicating the presence of CNTs in MOF/T. For MOF/H, an enhanced D-band is observed, resulting from the unique geometric structure and defects of CNHs, while the G-band remains unsplit, suggesting the presence of CNHs and the absence of CNTs. In contrast, the Raman spectra of MOF/HT show both D- and G-bands with equivalent intensities, and the G-band is split. These results confirm the coexistence of CNHs and CNTs in MOF/HT, demonstrating the successful integration of the CNHs and CNTs.

To further confirm the successful incorporation of the MOF onto the surfaces of the different carbon material substrates (CNH, CNT, and HT), FTIR spectroscopy was conducted. As shown in Figure 2c, the MOF supported on different carbon substrates exhibited identical characteristic peaks, which correspond to those of the MOF/carbon materials. The peaks at 1560 and 1614 cm^−1^ are attributed to the υ(C=C) stretching of the benzene ring from the organic ligand H_2_BDC. The broad peak centered at 3430 cm^−1^ is assigned to the N–H bond from PDA, and the characteristic peaks of asymmetric and symmetric COO^−^ at 1500 and 1386 cm^−1^ indicate the successful coordination between nickel ions and BDC ligands [27]. These results confirm the successful fabrication of the MOF on the surfaces of various carbon material substrates, including CNHs, CNTs, and HT.

The chemical compositions and valence states of the samples were further analyzed by XPS. As shown in Figure 2d–f, the XPS spectra of the as-prepared MOFs with different carbon-based substrates were identical. In the XPS survey spectra of MOF/HT, MOF/H, and MOF/T (Figures S3–S5), the presence of C, O, N, and Ni elements was observed, which originated from the PDA and NiMOF. This confirms the successful incorporation of PDA and the surface modification of the different carbon substrates by the MOF. The high-resolution Ni^2+^ spectra for the different MOF/carbon materials exhibit identical characteristic peaks (Figure 2). In the high-resolution Ni 2p spectra (Figure 2d–f), two characteristic peaks at 856.4 and 873 eV, corresponding to the 2p^3/2^ and 2p^1/2^ of Ni^2+^ in the MOF, were observed [27,29], confirming the successful integration of the MOF onto the CNH or CNT surfaces. These results further demonstrate that the active sites of the various MOF/carbon materials were identical.

#### 2.2.2. OER Performance of MOF/HT

**Figure 3:**
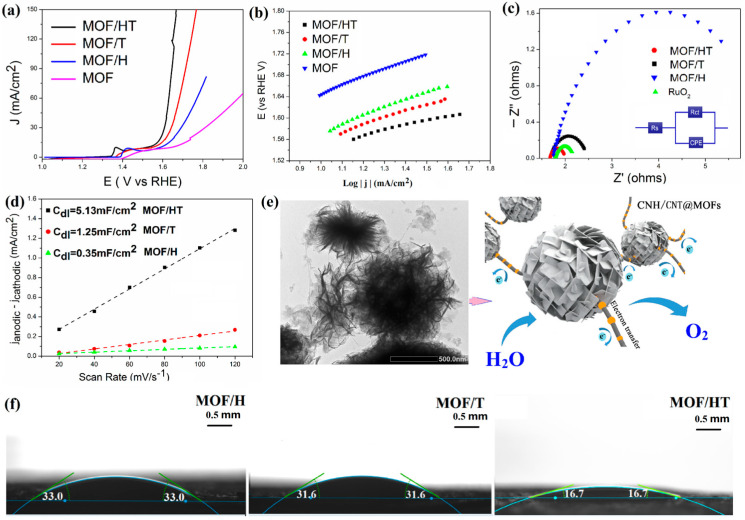
OER LSV curves (**a**); Tafel plots (**b**); EIS Nyquist plots of different MOF/carbon materials (**c**); charging current–density difference plotted against scan rate to yield double-layer capacitance (Cdl) of different catalysts (**d**); schematic of the electrocatalytic properties of MOF/HT (**e**); contact angles of water droplets on different samples at room temperature (**f**).

The OER activity of the as-prepared catalysts was assessed through linear sweep voltammetry (LSV) conducted with a CFP electrode that was modified with the electrocatalyst in a 1 M KOH solution. All the LSV data were adjusted to account for the ohmic drop (80% iR). As shown in Figure 3, the introduction of carbon-based substrates significantly lowered the overpotential required to achieve the same current density as that of the pure MOF. Among the different composites, MOF/HT, which featured a pseudo-neuronal structure, exhibited the lowest overpotential compared to MOF/H and MOF/T. Specifically, the overpotentials required to attain a current density of 10 mA cm^−2^ for the MOF, MOF/H, MOF/T, and MOF/HT were 392, 360, 320, and 280 mV, respectively. Notably, the MOF/HT catalyst displayed a reduction of 28% in the overpotential at 10 mA cm^−2^ compared to the pure MOF.

Tafel slopes, derived from the corresponding LSV curves, were employed to analyze the catalytic kinetics and provide insights into the OER mechanism. The values for the Tafel slopes of MOF/H, MOF/T, and MOF/HT were found to be 93, 83, and 80 mV·dec^−1^, respectively. Among these, MOF/HT exhibited the smallest Tafel slope, which suggests that it facilitates the fastest catalytic reaction. This result aligns with the findings from the LSV analysis.

Based on the previous characterization, it is evident that the active sites of MOFs supported on various carbon-based barrier catalysts remain consistent. However, distinct variations in the OER performance of these catalysts were observed. This discrepancy can be attributed to structural factors. As indicated by the SEM and TEM analyses, MOF/HT exhibited a neuron-like structure, which offers an increased number of reaction interfaces for catalytic processes. Additionally, the ‘cyton’ CNH@MOF transitioned into an Echinoida-like morphology, further exposing more active sites for the OER. The extensive network of CNTs, resembling synapses, facilitates enhanced electron transport during the OER process, which in turn contributes to the superior OER performance of the catalyst.

This was further supported by the electrochemically active surface area (ECSA), which reflects the effective area of the catalyst involved in the OER and is typically assessed by measuring the double-layer capacitance (Cdl) in a Faradaically silent region, as they exhibit a positive correlation. As illustrated in Figure 3d, MOF/HT demonstrated larger Cdl, suggesting a greater ECSA. This indicates that MOF/HT, with its distinctive Echinoida-like structure, exposed more active sites for participation in the OER process, ultimately leading to enhanced catalytic performance. 

Contact angle measurements serve as a key indicator of surface characteristics, particularly wettability. A lower contact angle signifies better wettability. The contact angles of deionized water on the surfaces of various prepared samples at room temperature are shown in Figure 3f. The recorded contact angles for MOF/H, MOF/T, and MOF/HT were 33.0°, 31.6°, and 16.7°, respectively. This trend demonstrates that the pseudo-neuronal structure facilitates reduced contact angles, enhancing the surface’s hydrophilic properties. As a result, the 3D pseudo-neuronal network plays a pivotal role in improving the hydrophilicity, ensuring an optimal interaction between the catalyst and the reactants, which consequently enhances the OER performance.

The introduction of a unique 3D substrate not only enhanced the ECSA and hydrophilicity, but also improved the electrical conductivity. EIS measurements were performed to examine the electrode kinetics at the interface between the catalyst and electrolyte during the oxygen evolution reaction (OER). Figure 3c presents a well-fitted curve corresponding to the modified Randles equivalent circuit. The series resistance (R_s_), observed at a high frequency, reflects the contact resistance between the catalyst and the current collector. Additionally, the charge transfer resistance (R_ct_), determined from the diameter of the semicircular arc, provides insights into the electron transfer and conductivity of the catalyst, which remains unaffected by the applied voltage. In this study, the R_s_ values for different samples were similar, while their R_ct_ values varied. As illustrated in Figure 3c, the incorporation of carbon-based barriers led to a reduction in R_ct_ compared to the pure MOF, with MOF/HT exhibiting the smallest R_ct_ compared to MOF/H and MOF/T. This observation confirms the enhanced charge transfer and conductivity for the OER, further suggesting that the pseudo-neuronal structure contributes to the improvement in the electrical conductivity. 

To summarize, the unique three-dimensional pseudo-neuronal network structure present in MOF/HT plays a crucial role in enhancing the exposure of active sites, improving the hydrophilicity, and increasing the electrical conductivity. As a result, MOF/HT demonstrates superior catalytic performance compared to both MOF/T and MOF/H, thanks to these structural advantages.

### 2.3. Preparation and Characterization of MOF/HT-S

**Figure 4:**
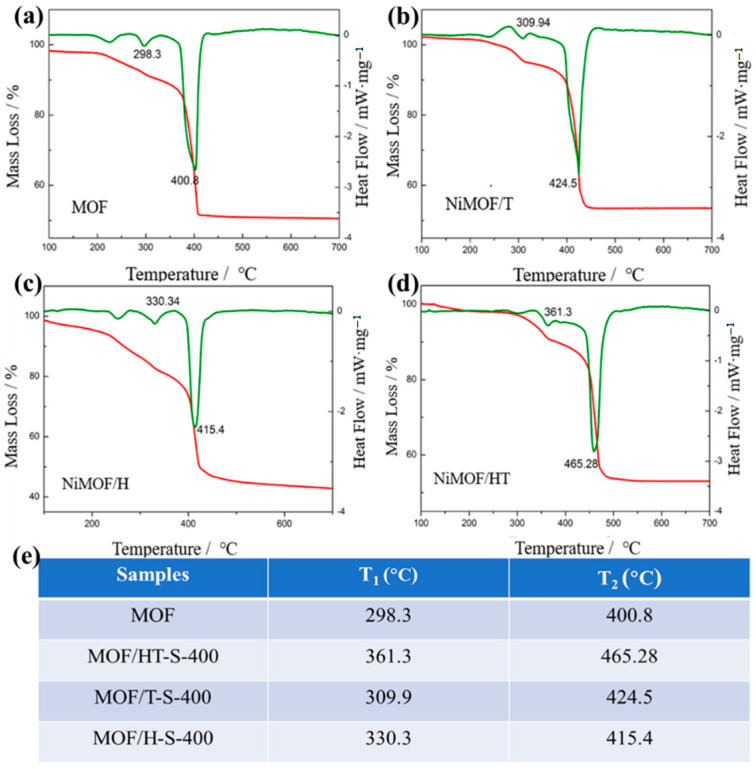
TG-DSC of MOF and MOF/carbon materials ((**a**–**d**), The red lines are the TG curves and the green lines are the heat dissipation curves); decomposition stages of different samples and their corresponding temperatures (**e**).

The composites formed by combining the MOF with TMSs retain the inherent properties of the MOF and can be synthesized through a controlled partial vulcanization process. Given the structural instability of MOFs and the tendency for TMS particles to aggregate, careful control over the vulcanization temperature and duration is essential for the successful fabrication of a TMS/MOF composite. The thermal analysis via TG/DSC (Figure 4) reveals that the decomposition of the MOF and MOF/carbon materials involves two distinct endothermic stages, which include the removal of guest molecules followed by structural collapse. It is evident that the temperature at which structural collapse occurs for MOF/carbon composites is notably higher than that of the pure MOF. Notably, MOF/HT demonstrates the highest structural collapse temperature among the MOF/carbon materials, highlighting its superior stability and suitability for the preparation of TMS derivative composites with MOFs.

Given the outstanding thermodynamic stability exhibited by the MOF/HT catalyst, which features a neuron-like structure, this material was employed as a template for the synthesis of a hierarchical electrocatalyst, NiS_2_ NPs/MOF/HT. The fabrication process involved a carefully controlled vulcanization treatment under specific atmospheric conditions.

#### 2.3.1. Characterization of MOF/HT-S

**Figure 5:**
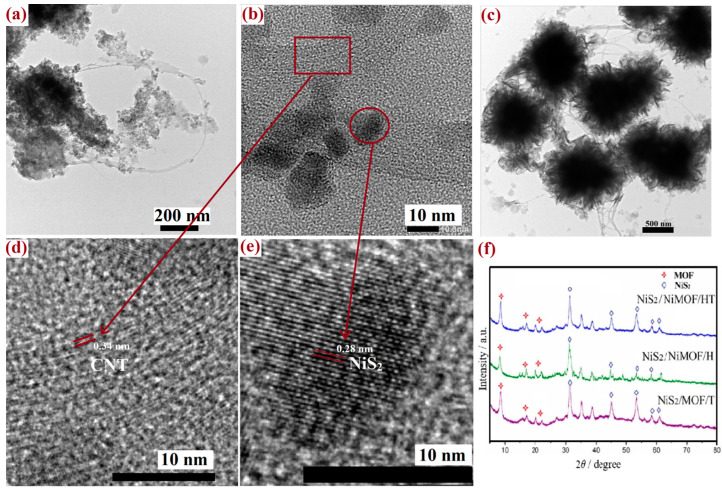
TEM image of MOF/HT-S (**a**,**b**,**d**,**e**) and MOF/HT (**c**); XRD of MOF/HT-S, MOF/T-S, MOF/H-S (**f**).

The phase transition of MOF/HT during the vulcanization process was analyzed using XRD. As depicted in Figure 5f, both NiS_2_ and MOF characteristic diffraction peaks were present in the XRD patterns of MOF/HT-S, confirming that the MOF structure did not undergo complete decomposition and that NiS_2_ was formed on the pseudo-neuronal HT structure. This indicated the successful fabrication of the hierarchical NiS_2_/MOF/HT composite. Moreover, similar diffraction peaks for both the MOF and NiS_2_ nanoparticles were observed in the XRD patterns of MOF/H-S and MOF/T-S. These results suggest that NiS_2_ nanoparticles and partially decomposed MOF/carbon-based materials (CNT, CNH, HT) were generated on the surfaces of the various carbon-based MOF composites following partial vulcanization. The active sites of all hybrid structure samples remained identical after the partial vulcanization process.

TEM was employed to examine the microstructures of the sulfide derivatives of MOF/HT. As illustrated in Figure 5, the original pseudo-neuronal structure was preserved, with spherical ‘cyton’ units interconnected by CNTs resembling synapses. Additionally, the Echinoidea-like morphology showed partial decomposition. Numerous particles were observed on the surface, yet the pseudo-neuronal arrangement remained intact. Further high-resolution TEM imaging (Figure 5d,e) confirmed that these particles were NiS_2_ nanoparticles. The lattice fringes observed in Figure 5d,e exhibited spacings of 0.28 and 0.34 nm, corresponding to the (210) planes of cubic-phase NiS_2_ and the graphitic characteristics of CNTs [29]. This observation further substantiates the coexistence of the NiS_2_ nanoparticles and partially decomposed MOF within MOF/HT-S, consistent with the findings from the previous XRD analysis.

**Figure 6:**
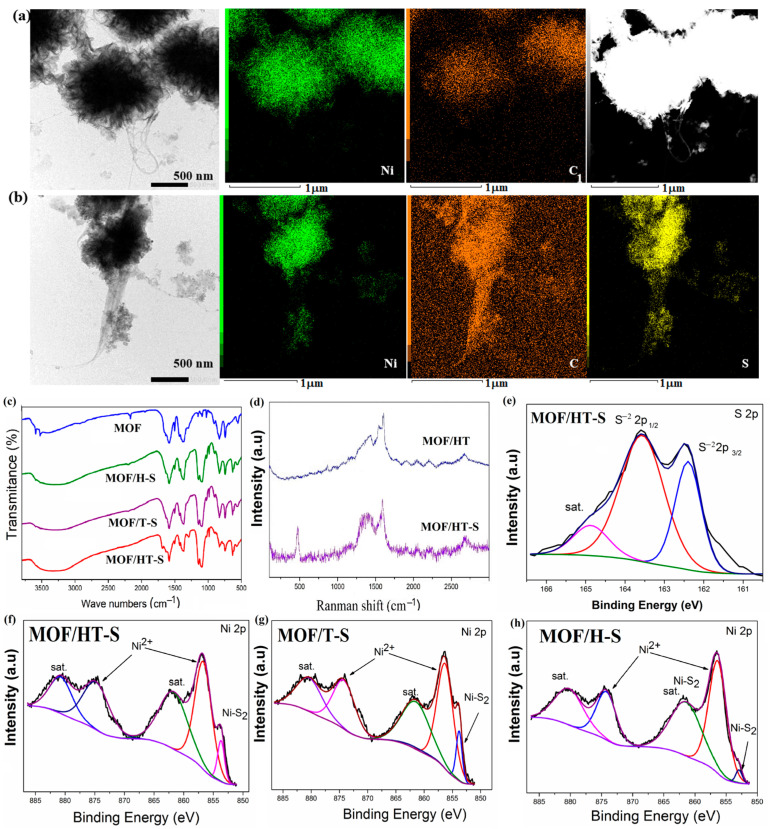
TEM image, the corresponding mapping, and STEM image of MOF/HT (**a**) and MOF/HT-S (**b**); FTIR spectrum (**c**); Raman spectrum (**d**); high-resolution spectra of S 2p (**e**) and Ni 2p ((**f**–**h**), The black curve is the experimentally measured data, and the color curve is the mathematical fit to it) of different as-prepared samples.

STEM mapping further supported the presence of sulfur in the sample. As shown in Figure 6a,b, elements such as C, N, O, and Ni were uniformly distributed within MOF/HT. Following partial sulfuration, S appeared in MOF/HT-S.

The formation of NiS_2_ was also validated by Raman spectroscopy. In the Raman spectra of MOF/HT-S (Figure 6d), a new peak at 470 cm^−1^ was observed, corresponding to the S-S stretching vibrations in NiS_2_, confirming its presence. Furthermore, the D- and G-bands of a similar intensity, along with the split G-band, were attributed to CNHs and CNTs, respectively. These features were clearly detected in the Raman spectrum of MOF/HT-S, indicating the presence of CNHs and CNTs in the composite.

An FTIR analysis was performed to determine the chemical composition of MOF/HT-S. The FTIR spectra (Figure 6c) reveal the symmetric and anti-symmetric vibration bands of the -COO- group at 1585 cm^−1^ and 1390 cm^−1^, respectively. Characteristic peaks corresponding to the -COOH group are seen at 1950 cm^−1^, and the typical C=C vibrations of the benzene ring skeleton are detected at 1443 cm^−1^ and 1502 cm^−1^, which are attributed to the MOF component. These findings confirm the presence of the MOF in MOF/HT-S and indicate the partial decomposition of MOF/HT. However, due to the effects of high-temperature vulcanization, the intensity of the -COO- peaks at 1585 cm^−1^ and 1390 cm^−1^ decreased, and the peaks associated with the -COOH group at 1950 cm^−1^ were no longer observed. Additionally, a peak corresponding to the S-S stretching vibrations was identified in the range of 1000–1200 cm^−1^, suggesting that the decomposed MOF/HT underwent in situ conversion into sulfides.

An XPS analysis was performed to determine the chemical composition of MOF/HT-S. The survey spectra indicated the presence of five elements in MOF/HT-S, namely C, O, N, Ni, and S (Figure S5). In the high-resolution S 2p spectrum (Figure 6e), the peaks observed at 162.3 and 163.6 eV correspond to the S_2_^2−^ 2p_3/2_ and S_2_^2−^ 2p_1/2_ states in NiS_2_ [29,30], confirming the formation of NiS_2_ within the MOF/HT-S composite. Additionally, the Ni 2p high-resolution spectra (Figure 6f,g) show the primary peaks at 856.4 and 873 eV, which align with the binding energies of the 2p_1/2_ and 2p_3/2_ states of Ni^2+^ in NiMOF, thus verifying the retention of the MOF structure in the vulcanized product. A smaller peak at 853.6 eV can be attributed to Ni^2+^ from NiS_2_. These findings confirm the coexistence of both the MOF and sulfide phases in the sulfurized MOF/HT derivatives.

In the high-resolution XPS spectra of Ni 2p and S 2p for MOF/H and MOF/T, distinct peaks corresponding to Ni^2+^ and (S_2_)^2−^ were observed. These findings further support the simultaneous presence of NiS_2_ and the MOF in the samples.

#### 2.3.2. OER Performance of MOF/HT-S

**Figure 7:**
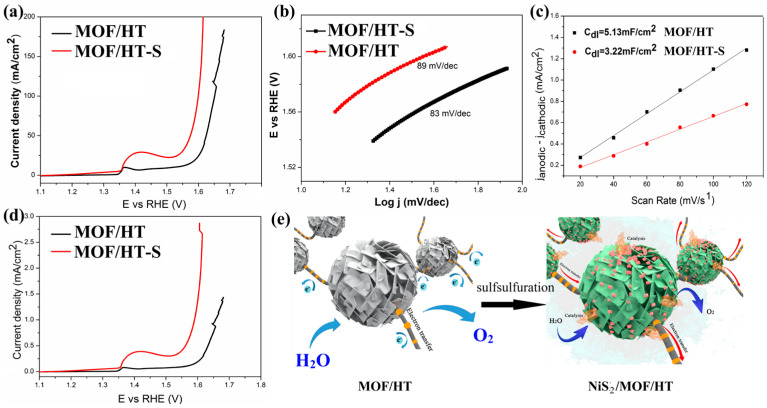
OER LSV curves (**a**); Tafel plots (**b**); charging current–density difference plotted against scan rate to yield double-layer capacitance (Cdl) of MOF/HT-S and MOF/HT (**c**); LSV curves normalized to ECSA (**d**); schematic of the electrocatalytic properties of MOF/HT and MOF/HT-S (**e**).

**Figure 8:**
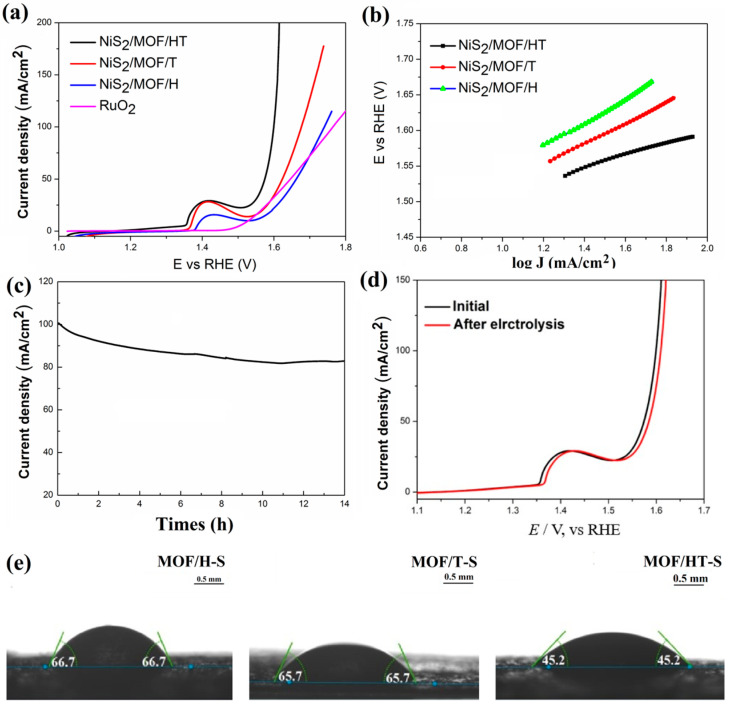
OER polarization curves (**a**); Tafel plots (**b**); chronoamperometry curves at 100 mA/cm^2^ (**c**); LSV curves of MOF-HT-S before and after stability test (**d**); contact angles of water droplets on different samples at room temperature (**e**).

In this study, the partial vulcanization of MOF/HT-S resulted in significantly enhanced OER activity, demonstrated by a 296 mV overpotential at a current density of 25 mA cm^−2^ (Figure 7a), which represents a 19.5% reduction compared to MOF/HT (368 mV). Furthermore, MOF/HT-S outperformed the commercial catalyst RuO_2_ at higher current densities (Figure 8a). Additionally, the Tafel slope of MOF/HT-S was smaller than that of MOF/HT, suggesting improved OER kinetics for the former.

The difference in catalytic performance between MOF/HT and MOF/HT-S was further confirmed by analyzing the ECSA of both materials. As illustrated in Figure 7c, the ECSA of MOF/HT-S is smaller than that of MOF/HT, which can be attributed to the partial collapse of the MOF structure during the high-temperature vulcanization process. A reduced ECSA suggests that fewer active sites are available for the OER in MOF/HT-S; however, it still exhibits a lower overpotential compared to MOF/HT. This observation highlights the superior intrinsic catalytic activity of MOF/HT-S, which can be ascribed to the synergistic interaction between NiS_2_ and the MOF structure [27].

This conclusion was further supported by the polarization curves, which were normalized based on the corresponding ECSA. As shown in Figure 7d, MOF/HT-S required a lower overpotential to achieve the same current density as MOF/HT. The superior OER performance of MOF/HT-S compared to MOF/HT further validates its enhanced intrinsic catalytic activity. Based on prior analyses and characterizations, the surface of MOF/HT-S contains not only a partially decomposed MOF film layer but also NiS_2_ nanoparticles formed by in situ vulcanization at elevated temperatures. The combination of NiS_2_ nanoparticles, the MOF membrane, and the HT carbon matrix with a neuron-like structure creates a synergistic effect, which contributes to the improved OER performance of MOF/HT-S compared to the pre-sulfided MOF/HT catalyst.

Further evaluation of the OER performance of the composites containing NiS_2_ and MOF, each with distinct carbon-based barriers, was conducted using LSV and Tafel curves. As illustrated in Figure 8a, the overpotential required by MOF/HT-S to achieve the same current density is lower than that of MOF/T-S and MOF/H-S, demonstrating superior OER catalytic performance. A comparison of NiSx-based materials with other state-of-the-art catalysts is summarized in Table 1. The MOF/HT-S catalyst synthesized in this study outperforms the pure metal sulfide catalysts reported in the literature. Regarding the reaction kinetics, the Tafel slopes of MOF/HT-S, MOF/T-S, and MOF/H-S are 89, 118, and 135 mV·dec^−1^, respectively. The smaller Tafel slope of MOF/HT-S suggests that this catalyst exhibits more favorable kinetics during the OER process.

The wettability test revealed the contact angles of water droplets on MOF/HT-S, MOF/T-S, and MOF/H-S, which were measured to be 45.2°, 66.7°, and 65.7°, respectively. These results suggest that the pseudo-neuronal structure contributes to lower contact angles, enhancing the hydrophilicity of the surface. Consequently, the three-dimensional pseudo-neuronal network plays a crucial role in determining the material’s hydrophilic properties, which in turn affect its OER performance. 

Electrochemical impedance spectroscopy (EIS) was employed to assess the electrode kinetics at the catalyst/electrolyte interface during the OER. The R_s_ values for MOF/HT-S, MOF/T-S, and MOF/H-S were relatively similar, indicating effective ohmic contact between the catalyst and the substrate, CFP (Figure S6). However, MOF/HT-S exhibited the smallest Rct value, suggesting that it facilitates faster charge transfer and promotes a higher reaction rate during the OER process.

Chronoamperometry was applied at a steady potential to assess the long-term stability of the MOF/HT-S catalyst. As illustrated in Figure 8c, there was a negligible decrease in the current over time. An analysis of the electrode’s polarization curve following 14 h of electrolysis revealed minimal alterations in the catalyst’s activity, confirming the robust durability of MOF/HT-S during extended electrolytic operations.

In conclusion, catalysts exhibiting neuron-inspired architectures demonstrate superior catalytic efficiency. The formation of NiS_2_ enhances the active centers for catalysis. The simultaneous presence of NiS_2_ nanoparticles and MOFs leads to better OER capabilities than those observed with MOFs alone. Furthermore, substrates that mimic neuronal structures contribute to enhanced electrical conductivity. The synergy between these enriched catalytic sites and conductive substrates yields robust catalytic performance, as depicted in Figure 7e.

## 3. Experimental Section

### 3.1. Preparation of HT

Firstly, 25 mg of dopamine was dissolved in 100 mL of deionized water and magnetically stirred for 30 min. Then, 25 mg of CNHs and 12.5 mg of CNTs were simultaneously dispersed in the dopamine solution by sonication for 30 min. A tris solution was subsequently added to the mixture and it was magnetically stirred for 6 h. During stirring, an appropriate amount of hydrochloric acid was added to maintain the pH of the solution at 8.5. Finally, the as-prepared products were collected by washing and centrifuging them several times with deionized water; then, they were dried in an oven.

### 3.2. Preparation of MOF/HT, MOF/H, and MOF/T

To prepare MOF/HT, the obtained HT was added to a Ni (NO_3_)_2_·6H_2_O solution to bind the transition metal ions to the surface of the HT. After stirring for 30 min, the mixture was filtered and dried to obtain the solid product HT-Ni^2+^. Next, 0.059 g of HT-Ni^2+^, 0.87 g of Ni (NO_3_)_2_·6H_2_O, and 0.167 g of H_2_BDC were dissolved in 75 mL of DMF, followed by ultrasonic dispersion for 10 min. The resulting mixture was then transferred to a hydrothermal reactor for solvothermal reactions at 130 °C for 24 h. The final product was obtained after washing it three times with a methanol solution and freeze-drying it under a vacuum, yielding MOF/HT.

MOF/CNH (denoted as MOF/H), MOF/CNT (denoted as MOF/T), and the MOF were prepared following the same procedure, with the only difference being the type of added carbon material. CNHs were incorporated into the preparation of MOF/H, while CNTs were used in the preparation of MOF/T. No carbon material was added during the preparation of the pure MOF. 

### 3.3. Preparation of Sulfide Derivatives of MOF/HT, MOF/H, and MOF/T

The typical fabrication process for the sulfide derivative of MOF/HT involved a simple and feasible atmospheric vulcanization method. First, 0.03 g of MOF/HT and 0.3 g of sublimed sulfur were placed at each end of a porcelain boat, which was divided into two unequal halves by a baffle. The porcelain boat was then placed in the center of a tubular furnace, which was heated to 400 °C for 10 min under an argon atmosphere. After heating, the MOF/HT-S was obtained by cooling the porcelain boat to room temperature within the furnace. The sulfide derivatives of MOF/H and MOF/T, denoted as MOF/H-S and MOF/T-S, were prepared following the same procedure, with the only difference being the use of MOF/H and MOF/T as the precursors, respectively.

### 3.4. Characterization

Transmission electron microscopy (TEM, JEM-2100, JEOL, Tokyo, Japan) and scanning electron microscopy (SEM, S-4800, Hitachi, Tokyo, Japan) were used to examine the morphologies of the samples. The crystal structure was analyzed by X-ray diffraction (XRD, Bruker, Billerica, MA, USA). The chemical structure and composition of the samples were determined by X-ray photoelectron spectrometry (XPS, Escalab250, Thermo Fisher Scientific, Waltham, MA, USA) and Fourier transform infrared (FTIR, Thermo Scientific, Waltham, MA, USA), respectively. The thermal stability of the samples was investigated by thermogravimetric analysis and differential scanning calorimetry (TG-DSC, Selbu, Germany) under an argon (Ar) flow at a heating rate of 5 °C min^−1^.

### 3.5. Electrochemical Measurements

The electrochemical catalytic performance in 1 M KOH was measured using a standard three-electrode system with a CS350 electrochemical workstation. A platinum (Pt) wire was used as the counter electrode, and a silver/silver chloride (Ag/AgCl) electrode (protected by a salt bridge) served as the reference electrode. The working electrode was a carbon fiber paper (CFP)-coated sample (see details in Section 2). The measured potential values were converted to the potentials of the reversible hydrogen electrode (RHE) using the following formula:

E(RHE) = E_Ag/AgCl_ + E_CE_^0^ + 0.059 pH,

where E_CE_^0^ = 0.1976 V at 298 K, and E_Ag/AgCl_ is the experimentally measured potential against the Ag/AgCl reference electrode.

## 4. Conclusions

This work introduces an innovative approach to fabricating catalysts that exhibit superior OER performance. Initially, a structure mimicking neuronal networks was crafted by linking CNHs with CNTs. This pseudo-neuronal framework served as a base for the deposition of MOFs, forming the MOF/HT catalyst, which demonstrated a 28% reduction in overpotential at a current density of 10 mA/cm^2^ compared to the pure MOF catalyst. Furthermore, the incorporation of NiS_2_ into the MOF/HT structure was achieved through partial sulfidation, enhancing the catalyst’s performance. The modified MOF/HT catalyst displayed a significant decrease in overpotential of 19.5% at 25 mA/cm^2^, attributed to the synergistic effects of the NiS_2_ and the partially decomposed MOF structure.

## Figures and Tables

**Table 1:** Comparison of the OER performance of NiS_x_-based electrocatalysts in alkaline media.

Sample	η10	Substrate	Electrolyte	Ref.
MOF/HT	289	CFP	1.0 M KOH	This research
NiMOF/HT-S	180	CFP	1.0 M KOH	This research
Ni MOF(BDC)	480	Glassy carbon	0.1 M KOH	[31]
3D Gr/Ni-MOF	370	Glassy carbon	0.1 M KOH	[31]
NiS/NiS_2_	416	Glassy carbon	1.0 M KOH	[32]
MOF-derived Fe–NiS_2_@g-C_3_N_4_ composite	280	Glassy carbon	1.0 M KOH	[33]

## Data Availability

The data that support the findings of this study are available from the corresponding author upon reasonable request.

## Supplementary Materials

The following supporting information can be downloaded at https://www.mdpi.com/article/10.3390/molecules30010080/s1. Figure S1: TEM image for MOF/H; Figure S2: XPS survey spectra for NiMOF/HT; Figure S3: XPS survey spectra for NiMOF/T; Figure S4: XPS survey spectra for NiMOF/H; Figure S5: XPS survey spectra forMOF/HT-S; Figure S6: EIS Nyquist plots of different MOF/carbon materials.
