# Supplementary material for: Constructing Neuron-like Structured NiS2/MOF Composites with Enhanced Regulation of Electron Transport and Active Sites for Oxygen Evolution
Source: Molecules. 2024 Dec 28;30(1):80. doi: 10.3390/molecules30010080 (PMC11721963; doi:10.3390/molecules30010080)
Supplement: Supplementary file 1 [file molecules-30-00080-s001.zip › molecules-3368937-supplementary.pdf]

Constructing Neuron-like structured NiS_2/MOF composites with enhanced regulation of electron transport and Active sites for Oxygen Evolution

Yanli Guo [a], Di Zhou [a], Yanyan Huang[a*], iaolong Song[b], He Wei [c],*

a, School of Mechanical Engineering, Chengdu University, Chengdu 610106, China

b, State Key Laboratory for Mechanical Behaviour of Materials, Xi'an Jiaotong University, Xi'an 710049, China

c, School of Aeronautics and Astronautics, Sichuan Univeristy, Chengdu, 610065, China

1 Experiment

1.1 Chemicals and materials.

Dopamine hydrochloride (DOPA-HCl) (98%), 1,4-benzenedicarboxylate (H_2BDC), nickel nitrate hexahydrate ($Ni(NO_3)_2{\cdot}6H_2O$), CNT and elemental sulfur powders (S) were provided by Sigma–Aldrich. Dimethylformamide (DMF) was purchased from a Beijing Chemical Reagent company and used without further purification. CNHs were prepared by direct-current (DC) arc discharge with a positive pressure at 0.1 MPa.

1.2 Preparation of CNH

CNH was prepared by DC arc discharge with a positive pressure at 0.1M Pa. In the arc-discharge process, the current is 110 A and the voltage is 30 V. The cathode and anode are made of high-purity graphite rods with a purity o f up to 99.99%.

1.3 **Preparation of MOF**

To prepare MOF, the Ni(NO3)2 • 6H2O, H2BDC were dissolved in DMF , followed by ultrasonic dispersion at room temperature for 10 min. Finally, th e mixture was transferred to a hydrothermal reactor for solvothermal reactions

at 130° C for 24 h. The resulting product was obtained after being washed wi th methanol solution thrice and freeze-drying in a vacuum.

1.4 Electrochemical Test

A standard three-electrode system using a CS350 electrochemistry worksta tion was conducted to measure the electrochemical catalytic performance in 1M KOH. A platinum (Pt) wire was used as the reference electrode, and a silver/ silver chloride (Ag/AgCl) electrode (protected by a salt bridge) served as the c ounter electrode. Linear sweep voltammogram (LSV) was measured to evaluate the activity of the catalyst and all the polarization curves were corrected for an ohmic drop (80% iR). Electrochemical Impedance Spectroscopy (EIS) measu rements were conducted from 10^5 to 10^{-1} Hz with an AC amplitude of 0.01 V and the EIS data were fitted to Randles circuit. Chronopotentiometry was use d to study the stability.

The working electrode was a carbon fibre paper (CFP)-coated sample pre pared as follows: a sample (5 mg) and a nafion solution (5%, 8μL) were adde d to a 300-μL ethanol solution and sonicated for 30 min; catalyst ink was dro pped on the CFP (1 cm × 1 cm) surface and dried at room temperature for 1 2 h. Catalyst loading was as similar in quality as possible (~0.25 mg cm−2).

2 Results and discussion

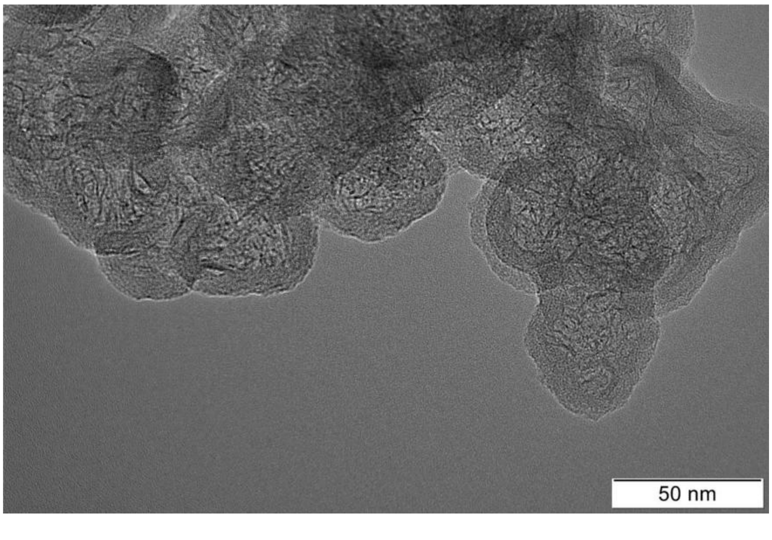

Figure S1. TEM image for MOF/H

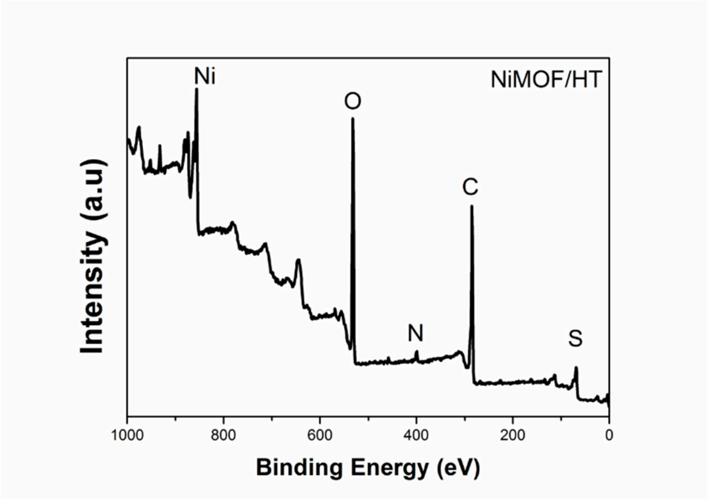

Figure S2. XPS survey spectra for NiMOF/HT

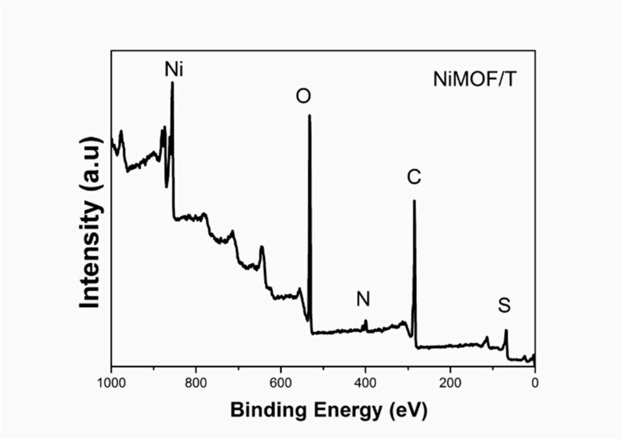

Figure S3. XPS survey spectra for NiMOF/T

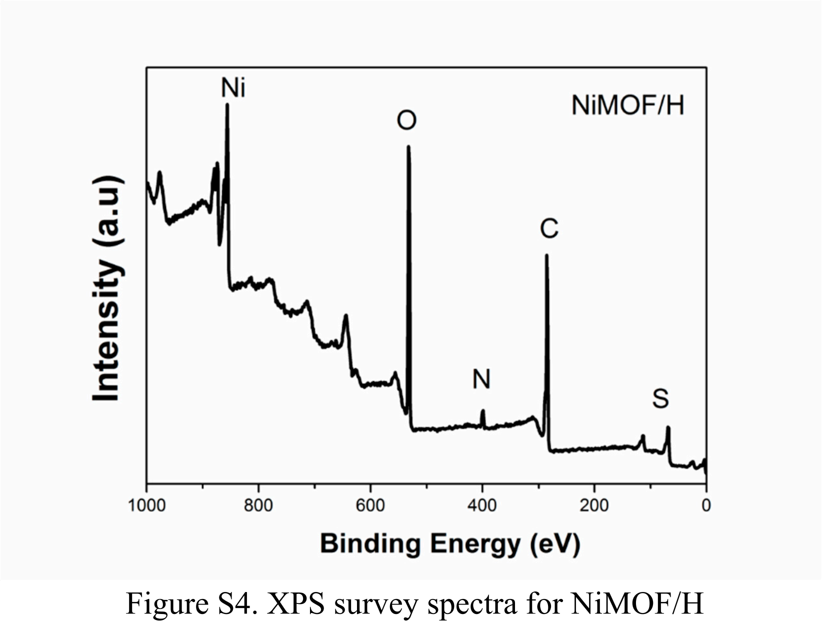

Figure S4. XPS survey spectra for NiMOF/H

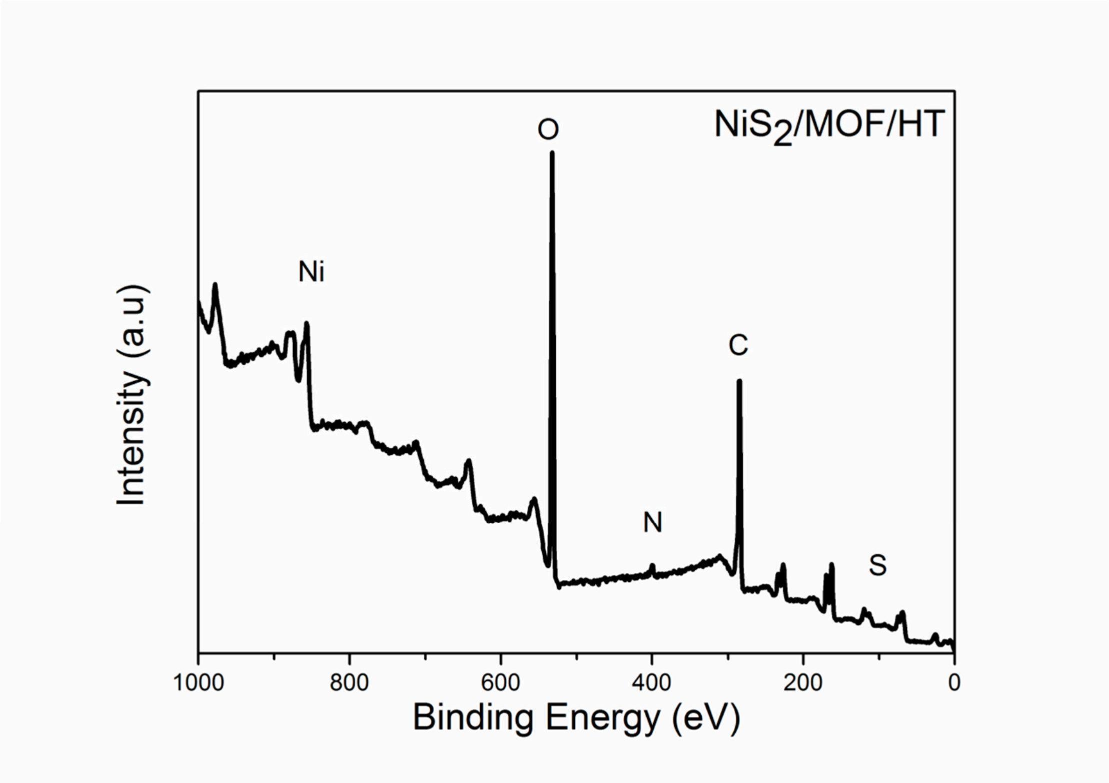

Figure S5. XPS survey spectra for MOF/HT-S

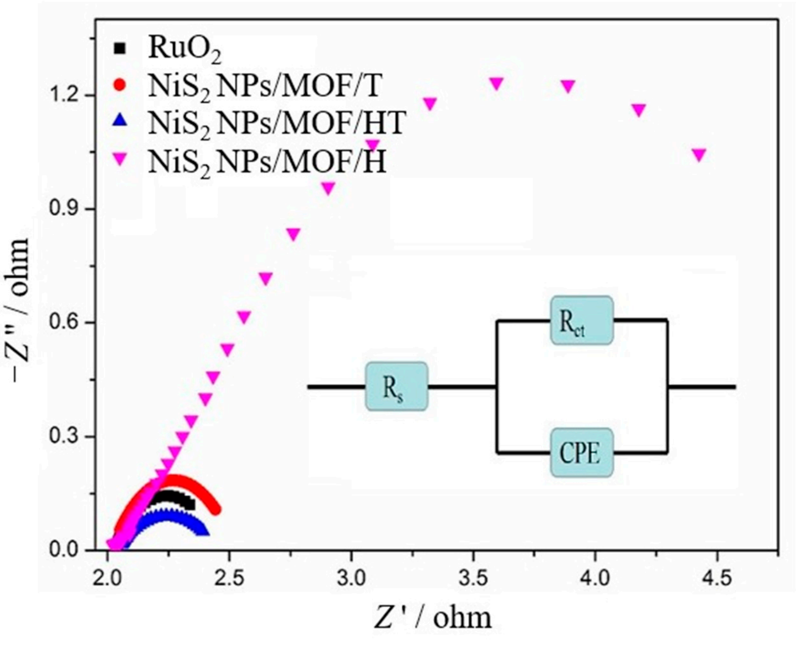

Figure S6. EIS Nyquist plots of different MOF/carbon materials
